# Measuring adoption of industry 4.0 technologies via international trade data: insights from European countries

**DOI:** 10.1007/s40812-021-00204-y

**Published:** 2022-01-10

**Authors:** Davide Castellani, Fabio Lamperti, Katiuscia Lavoratori

**Affiliations:** 1 Henley Business School, University of Reading, Whiteknights, RG6 6UD - Reading UK; 2 Department of Economics, University of Perugia, Via A. Pascoli, 20, 06123 Perugia, Italy

**Keywords:** Industry 4.0, Advanced manufacturing technologies, Technology diffusion, Industrial robots, Additive manufacturing, Industrial internet of things, COVID-19, F1, F2, O33, O34, O52

## Abstract

The investigation of the adoption of Industry 4.0 (I4.0) technologies and its implications, both at the macro and micro level, has attracted growing interest in the recent literature. Most studies have looked at the production and diffusion of related innovations and knowledge, but what do we know about the adoption of these technologies over time and across countries? In this paper, we look at three I4.0 technologies and present a new empirical perspective able to overcome the limitations of existing attempts at measuring their adoption, generally based on small-scale and country-specific studies. Our study provides a methodology that allows measuring adoption across countries for a relatively long time period. In so doing, we build on the well-established idea in the international economics literature that *trade* of capital goods captures technology diffusion, and so adoption across countries. We provide preliminary and comprehensive evidence on the adoption of these I4.0 technologies in Europe and set the premise for monitoring its evolution and implications on a large scale and over time.

## Introduction

While there is no universal agreement about what an industrial revolution is, there is consensus that three major technological shocks had a substantial impact on the way goods were manufactured throughout history. That is, the introduction of water and steam-powered manufacturing facilities; the electrically-powered technologies enabling mass production; the introduction of Information and Communication Technology (ICT) in the manufacturing process. More recently, governments, industries and academic scholars have highlighted the emergence of a new set of digital (and *smart*) technologies as the key players of a fourth industrial revolution (4IR) wave, also called Industry 4.0 (Brynjolfsson & McAfee, 2014; Davies, 2015; Schwab, 2016; OECD, 2017; UNCTAD, 2020; WIPO, 2019).^1^

Within the industrial manufacturing domain, the term *Industry 4.0* was coined in 2011 by the German Government to embrace the challenge of revitalising the manufacturing industry and boosting prosperity among developed economies, driven by the adoption and integration of a set of enabling advanced technologies (Kagermann et al., 2013; Mariani & Borghi, 2019; Rüßmann et al., 2015). In this paper, we will refer to *advanced manufacturing technologies* (AMTs) as this group of key player technologies driving such changing environment. AMTs are defined as “*computer-controlled or micro-electronics-based equipment used in the design, manufacture or handling of a product*” (OECD, 2012).

These technologies are seen as able to enhance operational flexibility, production efficiency and quality, and to reduce set-up costs, and so in turn to boost productivity and performance (Büchi et al., 2020; Rüßmann et al., 2015; Schwab, 2016; Skilton & Hovsepian, 2017), and create the conditions for sustainable operation management among supply chain operators (Lopes de Sousa Jabbour et al., 2018). In addition, they also allow for flexibility and speed in prototyping and responding to unpredictable demand needs and uncertainty. This has become extremely important since consumer needs and, more generally, the economic external environment are more and more volatile. Indeed, the role of technologies under the recent unprecedented circumstance of the COVID-19 pandemic is an inspiring example (The Guardian, 2020; European Commission, 2020; UNCTAD, 2020).

Despite the growing popularity of the matter across policy institutions, media and academic scholars, a clear picture of the adoption of I4.0 technologies on the global economy is still an under-investigated research area. Some evidence is provided using data collected from surveys in specific countries, or looking at specific technologies or on a small number of firms, through case studies (Dachs et al., 2019; Delic & Eyers, 2020; Sandström, 2016, among others). The main motivation for such paucity of evidence is the lack of reliable and precise measures of adoption on a large scale across countries and over time.

Some studies have looked at the production and diffusion of innovation and knowledge associated with the 4IR (Balland & Boschma, 2021; Benassi et al., 2020; Corradini et al., 2021; Felice et al., 2021; Venturini, 2021), with special reference to the technological and geographical aspects of the origin and diffusion of I4.0 knowledge and innovations (Balland & Boschma, 2021; Ciffolilli & Muscio, 2018; Corradini et al., 2021; Martinelli et al., 2021). However, more effort is needed to enhance our understanding of the magnitude and evolution over time, geographical spread across countries and the presence of specialisation patterns in the adoption of I4.0 technologies. This becomes extremely important for understanding a relatively new phenomenon and to provide suggestions both for policymakers and managers that are dealing with such technological changes.

From a methodological perspective, tracking the growth and evolution of emerging technologies is particularly complicated since there are no available data, especially when the transformation is still ongoing and the technology is not mature. Our empirical approach addresses this problem by relying on the well-developed idea that cross-country technology transfer can occur via international trade of capital goods.

In a seminal work, Caselli and Coleman (2001) investigate the technology diffusion of computers in the 70s–80s. At that time, computers represented a revolutionary innovation and a direct measure of capital investments was not available on a large scale. As an embodied technology, computers are an ideal case of technology diffusion to investigate, and as the authors do remark “*technology diffusion takes place through imports of the equipment embodying the technology*” (Caselli & Coleman, 2001, p. 328). Inspired by their intuition, we measure adoption of AMTs with import flows of selected products and machinery that embody such technologies, and we corroborate this measure with the use of production data able to capture the component of adoption, related to domestically produced goods. The idea of using imports as a proxy of technology adoption and diffusion has been developed in the literature (e.g. Acemoglu & Restrepo, 2018; Caselli & Wilson, 2004), and since these technologies belong to complex and high value categories of capital goods, the problem concerning re-exporting activities of imports in the form of intermediate inputs is very unlikely (Bernard et al., 2015).

In a nutshell, our methodology consists of identifying the fine-grained (8-digit) product codes of capital goods related to advance industrial robots (AIRs), additive manufacturing (AM) and industrial internet of things (IIoT), i.e. the three capital-embodied AMTs will we focus on. Based on these product codes, we can quantify the adoption of these technologies for 28 European countries over the 2009–2018 period. Our evidence suggests that the most advanced European economies have been investing in these technologies over the years with different degrees and technology specialisation. Interestingly, we also uncover a growing presence of a cluster of Central and Eastern European countries as I4.0 adopters. Two reasons can explain this finding: first, national industrial policies are massively supporting the adoption of such technologies to sustain long-term international competitive advantages; second, the increasing participation of these countries in global value chains (GVCs) facilitates the multinational enterprise (MNE) transfer of sophisticated production technologies to their foreign subsidiaries through imports of capital goods or encourages local suppliers to adopt advanced technologies in their production processes.

The main contribution of this work resides in moving forward the conversation about the adoption of AMTs within the I4.0 context, by introducing and improving an empirical measure able to capture the phenomenon. We provide *prima facie* empirical evidence of the diffusion of AMTs across European countries over the period 2009–2018. At the same time, we provide a discussion about possible extensions of such methodology at the industry and firm level, alongside a further research agenda.

The paper is organised as follows. The next section briefly describes the advanced manufacturing technologies under investigation. Section 3 describes the data and the methodology employed to create the measure of adoption and to identify AMTs from trade data. Section 4 provides an empirical application of the proposed methodology illustrating the relevance, evolution and geographical diffusion of AMT adoption across European countries. Section 5 concludes, summarizing the main findings and proposing possible research directions.

## Defining advanced manufacturing technologies

As discussed in the previous Section, I4.0 gathers a heterogeneous set of technologies, bearing different levels of mutual complementarity as well as different degrees of relatedness with specific industrial operations. These underlying similarities and differences, together with the characteristics of each I4.0 technology, motivate our focus on those technologies that have the highest potential impact in advanced manufacturing processes. Keeping this as our starting point, we embrace the definition provided by the European Foundation for the Improvement of Living and Working Conditions, which identifies five ‘*game-changing technologies*’, namely, advanced industrial robots (AIRs), additive manufacturing (AM), industrial internet of things (IIoT), electric vehicles and industrial biotechnologies (Eurofound, 2018). As anticipated, this paper focuses on the first three technologies given their potential to impact significantly all manufacturing sectors to the core of their operations, being part of the I4.0 wave.

Moreover, these are embodied technologies, so that their adoption requires a physical installation of a specific type of capital equipment. This is a crucial distinction concerning other new digital technologies of the 4IR (e.g. artificial intelligence, machine learning, cloud computing, big data, etc.), whose physical component of the technology is usually standardised and multi-purpose (Foster-McGregor et al., 2019). In turn, this further intrinsic feature of the three AMTs we investigate makes them more appropriate for the methodology that we are devising in this paper.

### Advanced industrial robots (AIRs)

This category includes advanced industrial robots, which leverage high-level and dynamic programming (i.e. able to perform ‘*smarter*’ tasks) and enable more flexibility in production (Eurofound, 2018). Thanks to the falling cost of hardware and software experienced during the last decade, there has been a huge improvement in the technical features of industrial robotics. Advanced robots existing nowadays can perform a wider set of tasks compared to their predecessors, especially those requiring high flexibility and accuracy. The possibility of equipping robots also with advanced sensors and functionalities, and the potential for human–machine interactions has enabled their adoption to spread from traditional sectors of usage (e.g. automotive and electronics) to several others (e.g. agriculture and logistics).

### Additive manufacturing (AM)

The International Organization for Standardization (ISO) defines additive manufacturing as “*the general term for those technologies that based on a geometrical representation creates physical objects by successive addition of material*” (ISO, 2015). Currently, these technologies are used for various applications in the engineering industry, but also in other areas such as medicine, architecture, education, and several handcrafted segments (Wohlers Associates, 2014). This category includes highly flexible and adaptable machinery leveraging on digital production technique enabling reduced material consumption and wastes as compared to ‘*traditional*’ subtracting methods (Achillas et al., 2015; Atzeni & Salmi, 2012; Chekurov et al., 2018; Tuck et al., 2008), technically enhanced and highly customised products (Atzeni & Salmi, 2012; Diegel et al., 2010; Khorram Niaki & Nonino, 2017; Mellor et al., 2014; Petrick & Simpson, 2013), as well as fewer manufacturing steps, especially reducing assembly operations (Cuellar et al., 2018; Sandström, 2016; Singamneni et al., 2019; Weller et al., 2015). Additive manufacturing (also referred to as 3D printing) techniques work by following a reversed logic than traditional manufacturing processes (Attaran et al., 2017), adding or melting subsequent 2D layers of material to generate the final product. Already implemented in the production of plastic consumer products, aerospace and human prosthetics, additive manufacturing is increasingly adopted in other manufacturing sectors (EIB, 2019; Laplume et al., 2016; OECD, 2017).

### Industrial Internet of Things (IIoT)

The Industrial Internet of Things is used to identify the industrial specializations of the Internet of Things (IoT). The latter consists of “*a global infrastructure for the information society, enabling advanced services by interconnecting (physical and virtual) things based on existing and evolving interoperable information and communication technologies*” (ITU, 2012). This category includes wireless (and not) sensors, actuators, control and regulation systems, microchips and distributed systems such as Near Field Communication (NFC) chips, Radio-Frequency Identification (RFID) tags and Global Positioning Systems (GPS) (Atzori et al., 2010; Gubbi et al., 2013). IoT systems can be applied to several different contexts to create smart environments (e.g. smart cities, smart homes, smart factories, smart vehicles, etc.). Specifically, Industrial IoT refers to the creation of a digital environment in which (1) controlling machines (i.e. computers), (2) process machinery (e.g. ‘*traditional*’ automatic manufacturing stations, additive manufacturing machines and industrial robots) and (3) smart products (i.e. products incorporating an RFID tag, NFC chip, GPS or alike systems) are all connected. Hence, IIoT integrates a high-level processing and communication potential able to elaborate huge data amount, collected and transferred between each node of a widespread, seamless network (Atzori et al., 2010; Gubbi et al., 2013). In turn, this creates opportunities for enhanced working conditions, more flexible operations and digital integration along the value chain (Stock & Seliger, 2016; Wang et al., 2016a; b).

## Data and methodology

### Building measures of I4.0 technology adoption

So far, the empirical literature has been strongly limited by the absence of an extensive, precise and comprehensive measure of adoption to capture such a complex phenomenon, across technologies, across countries and over time.

In particular, some evidence comes from data collected through surveys in specific countries or looking at specific technologies. For instance, data collected by the European Investment Bank (EIB, 2019) and from Eurostat (Eurostat, 2021) provide cross-country insights from a representative sample of firms adopting various technologies of the 4IR—at the aggregate and sectoral level, respectively, at the same time providing only cross-sectional evidence. Conversely, survey data providing insight at a finer level—cross-country, sectoral, or even firm-level adoption—are available either for long time-series although focusing on single technologies (like industrial robots in the case of data from the International Federation of Robotics (IFR)) or for more technologies but on a shorter period (such as for data from the European Manufacturing Survey (EMS)^2^). Alternatively, several contributions have addressed the implications of adopting I4.0 technologies directly, through case studies based on specific sectors or a small number of firms (Khorram Niaki & Nonino, 2017; Sandström, 2016), by small-scale firm-based surveys (Delic & Eyers, 2020; Kianian et al., 2016), or by extrapolation from alternative sources (Ancarani et al., 2019). In turn, these limits associated with the existing data sources hamper the comparison across countries and sectors, as well as across technologies. We aim at overcoming such data and methodology limitations.

Drawing from Caselli and Coleman (2001), we create two measures as a proxy of adoption: first, we measure adoption by the *import* of AMT capital goods, using bilateral trade data at the finest level of disaggregated product classes. However, we acknowledge that imports may underestimate adoption in countries that have a large local production of AMTs. To assess the extent of this potential measurement issue, we also resort to a different measure of adoption, which we call *net consumption,* based on the formula: $net consumption=(production+import-export)$. In this way, we can account for both sources of capital investments determining adoption of AMTs, that is domestic and foreign production. This second measure is not available for all countries and technologies considered, as production data on goods embodying AMTs are in some cases missing or not reliable. Therefore, this measure is mainly used to validate our *import* measure of AMT adoption, which is more widely available (also outside the EU) and thus allow us to extend the application of this methodology.

After completing the data collection on trade and production information, we create these adoption proxies for each of the three AMTs we look at (i.e. AIRs, AM and IIoT). First, we compute *import* variables by creating three ‘*synthetic*’ measures computed as the sum of all 8-digit product codes relating to the same technology (as illustrated in detail in Sect. 3.3), for each country-year observation in our EU28 sample. Following the same logic as for the *import* variables, we build our second proxy measuring adoption (i.e. the *net consumption* variables) by combining import, export and production data for each AMT.

We finally adjusted values for PPP and converted them in constant 2011 USD using exchange rates and PPP conversion factors from Eurostat and the World Development Indicator (WDI) data set of the World Bank, respectively, so to allow for intertemporal and geographical comparison and to filter out cross-country differences in prices.

### Data

We rely on two main sources of data to generate measures of AMT adoption. First, we use highly disaggregated trade data collected from the Comext data set, available on the Eurostat website. Comext provides statistics on the value of goods traded between the EU28 member states (i.e. intra-EU trade) and traded by the EU member states with non-EU countries (i.e. extra-EU trade) (Eurostat, 2019). Goods are classified according to the Combined Nomenclature (CN) classification, which is based on the harmonised Commodity Description and Coding System (HS). The HS provides information up to the 6-digits level of commodity disaggregation, and then the CN builds on the HS by adding a further breakdown at the 8-digit level. This extension allows us to consider around 9,500 8-digit product codes, which are subject to annual revisions that ensure the CN to be up to date to changes in technology or patterns of international trade (Eurostat, 2019).^3^ As our interest lies in the identification of very specific capital goods associated with three technologies, the use of 8-digit disaggregated data provides the insight needed to identify with a sufficient deal of precision those product categories in which it is more likely that these AMTs are traded.

Second, we use production data from the PRODCOM data set (Eurostat, 2018) to provide further detail to our analysis and build a measure of *net consumption*. The PRODCOM data set provides information on the value of goods produced and sold in EU28 countries. Differently from the data reported in Comext, PRODCOM data follow the Classification of Products by Activity (CPA). As in the case of the CN classification, the CPA is revised every year and consists of around 3,900 products; hence, one CPA product may correspond to one or more CN goods (even though in the case of some product categories the CPA features a higher level of detail as compared to the CN). Furthermore, the CPA classification differentiates itself from the CN one as it is based on the NACE Rev.2 classification. This means that the first 4-digits of each product code in the CPA corresponds to the 4-digit sector in which the product is manufactured.

Both Comext and Prodcom databases also report data on quantities of 8-digit products, traded and produced. Though quantities would represent a more desirable measure as they are not affected by inflation dynamics or conversions for international and intertemporal comparison, our preferred measures are based on value data. There are two main reasons for this choice: first, quantities are frequently reported in different ways in the two data sets,^4^ thus not allowing for comparison on the quantities of all product categories we look at. Second, data on quantities present a high share of missing values in our country-year observations for many of the disaggregated product codes we consider. Hence, we decided to employ value data as they enable higher comparability across the two sets of data. Data were collected in 2019 using the latest HS classification (i.e. HS-2017) and the corresponding versions of the CN and CPA classifications as reference.

### Identifying AMTs via trade data

Our identification of the specific types of machinery, equipment and components related to AMTs starts from the analysis of several sources of information. In particular, we relied on (i) the relevant engineering literature both from the practitioner—for instance, the standard international terminology approved by ASTM International (2013) and ISO (2015) for AM technologies, concepts and definitions on IIoT provided by ITU (2012)—and an academic point of view; (ii) product catalogues of representative producing firms for AIRs, IIoT and AM,^5^ (iii) the World Customs Organisation (WCO) and (iv) Eurostat.^6^ From these sources, we were able to develop a list of keywords related to our AMTs of interest. This keyword-based approach has been widely used lately, and applied to different data sources—e.g. patents, business registers, scientific publications, trade and industrial records (Craglia et al., 2018; De Prato et al., 2019; Van Roy et al., 2019). The list of identified keywords is reported in Table 5 in Appendix B.

The identified keywords were then used to define an initial list of 25 8-digit CN product codes.^7^ We acknowledge that some of the technologies we focus on may be embedded also in other product classes not included in our shortlist. However, we adopt a conservative approach that allows us to consider only those product codes reporting a precise, coherent and unquestionable description, and to underestimate rather than overestimate the phenomenon. At the same time, the selected keywords might also lead to false-positive results or matches with product codes at a lower level of disaggregation (e.g. 6- or 4-digit codes). Hence, we performed a second stage of manual screening in which we exclude potential false-positive matches and identify the relevant 8-digit codes included in the less disaggregated categories matching with our keywords. More specifically, we focus on trade in capital goods of product codes included in the 4-digit CN codes 8463 (Machine tools for working metal or cermets, without removing material), 8471 (Automatic data-processing machines and units thereof […]), 8477 (Machinery for working rubber or plastics or for the manufacture of products from these materials), 8479 (Machines and mechanical appliances having individual functions), 8515 (Electric, laser or other light or photon beam, electron beam […] machines and apparatus for hot spraying of metals or cermets), 8517 (Apparatus for the transmission or reception of voice, images or other data, including apparatus for communication in a wired or wireless network […]), 8526 (Radar apparatus, radio navigational aid apparatus and radio remote control apparatus), 8542 (Electronic integrated circuits) and 9032 (Automatic regulating or controlling instruments and apparatus). The full list of product codes initially identified and the related descriptions are reported in Table 6 in Appendix B.

In the case of AIRs, our initial research brought to the identification of a single, main, code—since we do not aim at considering other forms of more traditional automation like non-robotics handling machines or conveyor belt systems. The other two cases present more challenges: specific codes for AM machines and IIoT devices do not yet exist in either the HS or the CN classifications. In the case of AM, the World Customs Organisation recognises the lack of a specific chapter in the HS classification encompassing these types of machinery, thus resulting in their categorisation being spread in several other product codes (Yuk, 2018). To the best of our knowledge, the identified codes are those most suitable to be used in practice and reflect the specific characteristics of the existing AM processes, as described above. The case of IIoT is even more challenging as the variety of devices is larger than in the case of AM, and cases of our focus goods being matched to a wider set of product categories greatly increases. Nonetheless, based on further validation discussed below, we believe the set of codes shortlisted here should capture much of the trade associated with IIoT components as product descriptions of the shortlisted goods refer to very specific products, classified in a highly detailed way.

To validate the selection process for the shortlisted CN codes, we first developed a survey to collect information on the CN (and/or CPA) product codes used by producers of the three AMTs when exporting (and/or producing) their products. Then, we consulted experts and practitioners from the Italian Customs Agency and a private customs broker. Overall, the 8-digit codes originally identified were confirmed and, at the same time, no initially shortlisted code was discarded, hinting to the goodness of the overall identification procedure. Appendix A provides further details on the validation process.

After the validation process, we matched the 25 CN codes considered in Table 6 with 26 codes in the CPA nomenclature, according to the 2017 correspondence table provided by Eurostat. Since a crucial task for our analysis lies indeed in the identification of the correct product codes associated with our AMTs, when looking at past and subsequent years, we use year-to-year correspondence tables provided by Eurostat, and first we checked for forward and backwards changes that occurred in each of the two classifications along the period considered (2009–2018); second, we cross-checked the correspondence between the CN and the CPA classifications year-by-year to track any potential change related to the identified codes.

Changes in the CN and CPA classifications are of two types: (1) new products are added to the classifications with new codes; (2) existing product codes are converted into new product codes. Changes of this second type are problematic, as they might imply not just the ‘recoding’ of certain products but also the elimination of ‘old’ product codes, whose related products are then absorbed in one (or more) new codes. Specifically, in cases in which multiple CN codes correspond to one or more CPA codes (or vice versa), as well as for cases in which the classification has changed over time, we followed the methodology by Van Beveren et al. (2012). This methodology proceeds by creating ‘*synthetic*’ codes by grouping together the codes which are subject to changes. In this way, we ensure full consistency in the correspondence between trade and production data over time.

When looking at the product codes we have identified as capturing AMTs, this procedure resulted in the reduction of our product codes from 25 to 22 following the CN nomenclature, and from 26 to 20 following the CPA nomenclature. Our cross-checking procedure highlighted a mostly consistent correspondence of the product codes, across both years and classifications, with only a few cases in our list of codes subject to either type (1) or type (2) changes. Table 1 reports the correspondence table between CN and CPA codes, in 2017.

**Table 1:** Correspondence between CN and CPA product codes related to AMTs

8-digits product codes CN	CPA product codes	CPA product descriptions
Advanced Industrial Robots		
84795000	28993935	Industrial robots for multiple uses (excluding robots designed to perform a specific function (e.g. lifting, handling, loading or unloading))
Additive Manufacturing		
84639000	28413471	Swaging machines and spinning lathes for working metal, machines for manufacturing flexible tubes of spiral metal strip and electro-magnetic pulse metal forming machines, and other machine tools for working metal without removing metal (excluding riveting machines)
	28491360	Riveting machines
	289900Z0	Riveting machines, swaging machines and spinning lathes for working metal, machines for manufacturing flexible tubes of spiral metal strip and electro-magnetic pulse metal forming machines, and other machine tools for working metal without removing metal
84772000	28961030	Extruders for working rubber or plastics, or for manufacturing rubber or plastic products
84775980	28961075	Machinery for moulding or forming rubber or plastics, etc., n.e.c
84778011	28961082	Machines for processing reactive resins
84778019	28961084	Machines for the manufacture of foam products (excluding machines for processing reactive resins)
84778099	28961097	Machinery for working rubber or plastics or for the manufacture of products from these materials, n.e.c
85158010	27903181	Electrical machines and apparatus for welding or spraying of metals, n.e.c
85158090	27903191	Electrical machines and apparatus for welding thermoplastic materials (excluding wire bonders of a kind used for the manufacture of semiconductor devices)
Industrial Internet of Things		
84718000	26122000	Network communications equipment (e.g. hubs, routers, gateways) for LANs and WANs and sound, video, network and similar cards for automatic data processing machines
84719000	26203000	Other units of automatic data processing machines (excluding network communications equipment (e.g. hubs, routers, gateways) for LANs and WANs and sound, video, network and similar cards for automatic data processing machines)
	269900Z0	Other units of automatic data processing machines
85176200	26302320	Machines for the reception, conversion and transmission or regeneration of voice, images or other data, including switching and routing apparatus
85269120	26512050	Radio navigational aid apparatus (including radio beacons and radio buoys, receivers, radio compasses equipped with multiple aerials or with a directional frame aerial)
85269200	26512080	Radio remote control apparatus (including for ships, pilotless aircraft, rockets, missiles, toys, and model ships or aircraft, for machines, for the detonation of mines)
85423111	26113003	Multichip integrated circuits: processors and controllers, whether or not combined with memories, converters, logic circuits, amplifiers, clock and timing circuits, or other circuits
85423119		
85423190	26113006	Electronic integrated circuits (excluding multichip circuits): processors and controllers, whether or not combined with memories, converters, logic circuits, amplifiers, clock and timing circuits, or other circuits
85423911	26113091	Other multichip integrated circuits n.e.c
85423919		
85423990	26113094	Other electronic integrated circuits n.e.c
90321020	26517015	Electronic thermostats
90321080	26517019	Non-electronic thermostats
90322000	26517030	Manostats
90328100	26516500	Hydraulic or pneumatic automatic regulating or controlling instruments and apparatus
90328900	26517090	Instruments and apparatus, regulating or controlling, n.e.c

The reference CN and CPA classifications are 2017 versions

## Discussion of finding

In this Section, we present the main trends over time and across countries characterising the diffusion of AIRs, AM and IIoT across EU28 countries, between 2009 and 2018. The choice of focussing on the period after 2009 is driven by the following considerations. In 2006 the German government has launched the High-Tech Strategy to drive innovation actions and technological innovation. In 2009, after the global financial crisis, the demand for mechanical engineering products returned to normal (Kagermann et al., 2013). In the same year, Korea has launched a five-year plan to encourage R&D investments in the intelligent robot industry aiming at expanding the adoption of industrial robots in other industrial sectors, since industrial robotics can be considered the first key technological driver (De Backer et al., 2018). Furthermore, several core patents protecting additive manufacturing technologies, such as fused deposition modelling and selective laser sintering, expired between 2009 and 2014 (Laplume et al., 2016). This created the right conditions for many new producers of additive manufacturing machinery to start their business about spill-over inventions (Wohlers Associates, 2014). Thus, we start the period of observation from 2009, which can be reasonably recognised as the beginning of a global ferment on this technological wave.

### Preliminary insights on AMT adoption

Our first focus is on the relationship between *import* and *net consumption* measures in our EU28 sample, over the 2009–2018 period. This relationship can be explored only on the subsample of countries for which production data are available for the product codes described in Sect. 3; hence, for which *net consumption* can be computed.

Rooting our argument in the literature on technology diffusion (e.g. Acharya & Keller, 2009; Caselli & Coleman, 2001; Caselli & Wilson, 2004),^8^ we argue that *import* represents a good proxy of AMT adoption, especially for those countries not characterised by a strong local production for such technologies. Conversely, when local producers account for a substantial share of adoption, the *net consumption* proxy should provide more precise insights into the phenomenon.

**Fig. 1:**
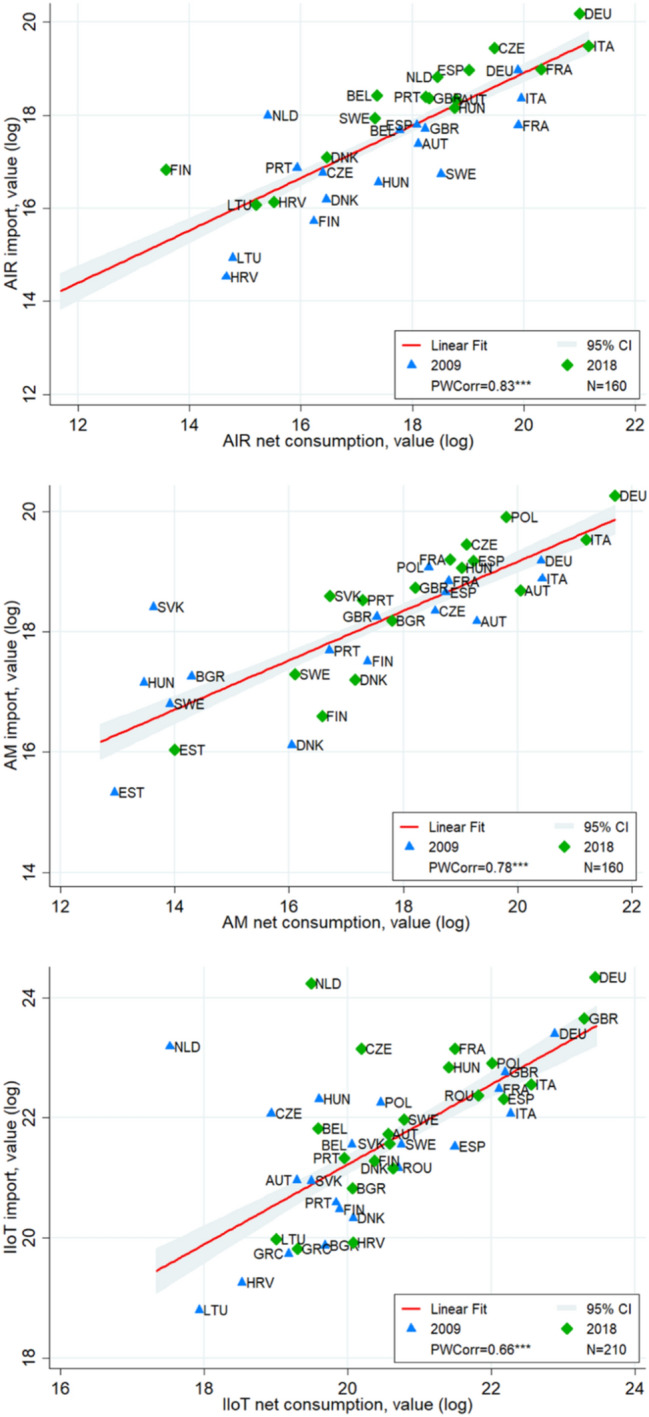
Relationship between *import* and *net consumption* measures of AMT adoption, 2009 and 2018 values and pairwise correlation coefficients. *Import* and *net consumption* measures converted in constant PPP USD to increase comparability over time and filter out cross-country differences in prices. Source: Comext and PRODCOM databases

Figure 1 plots values of our two adoption proxies at the beginning and the end of the observation period, showing that *import* and *net consumption* are highly correlated, with pairwise correlation coefficients of 0.83 for AIR, 0.78 for AM and 0.66 for IIoT. With the exception of The Netherlands in IIoT, where import is much larger than net consumption, probably due to the export of imported components, import and net consumption largely coincide for all three AMTs. Indeed, the Figure reveals our two measures to be largely comparable across European countries for which we have production data—because the net difference between production and export of AMTs is negligible in the case of most countries and technologies—and *import* to be an almost perfect measure of adoption.

Despite some differences across the three technologies, this first descriptive evidence suggests that *import* can be a good proxy of adoption for AMTs across EU countries.

Furthermore, looking at the relative positioning of most EU28 countries in the initial and final year in our sample highlight a proportional change in both *import* and *net consumption* proxies. This suggests that the large majority of European countries have been increasingly adopting AMTs. In the following, we argue that this measure indeed captures the patterns of adoption over time and across countries.^9^

### Temporal and geographical patterns of AMT adoption

As discussed in the previous Sections, these technologies have received considerable attention from businesses and policymakers, and they have been at the core of several industrial initiatives worldwide after the 2009 financial crisis. Hence, we expect the adoption of AMTs across EU28 countries to have significantly increased over our observation period.

**Fig. 2:**
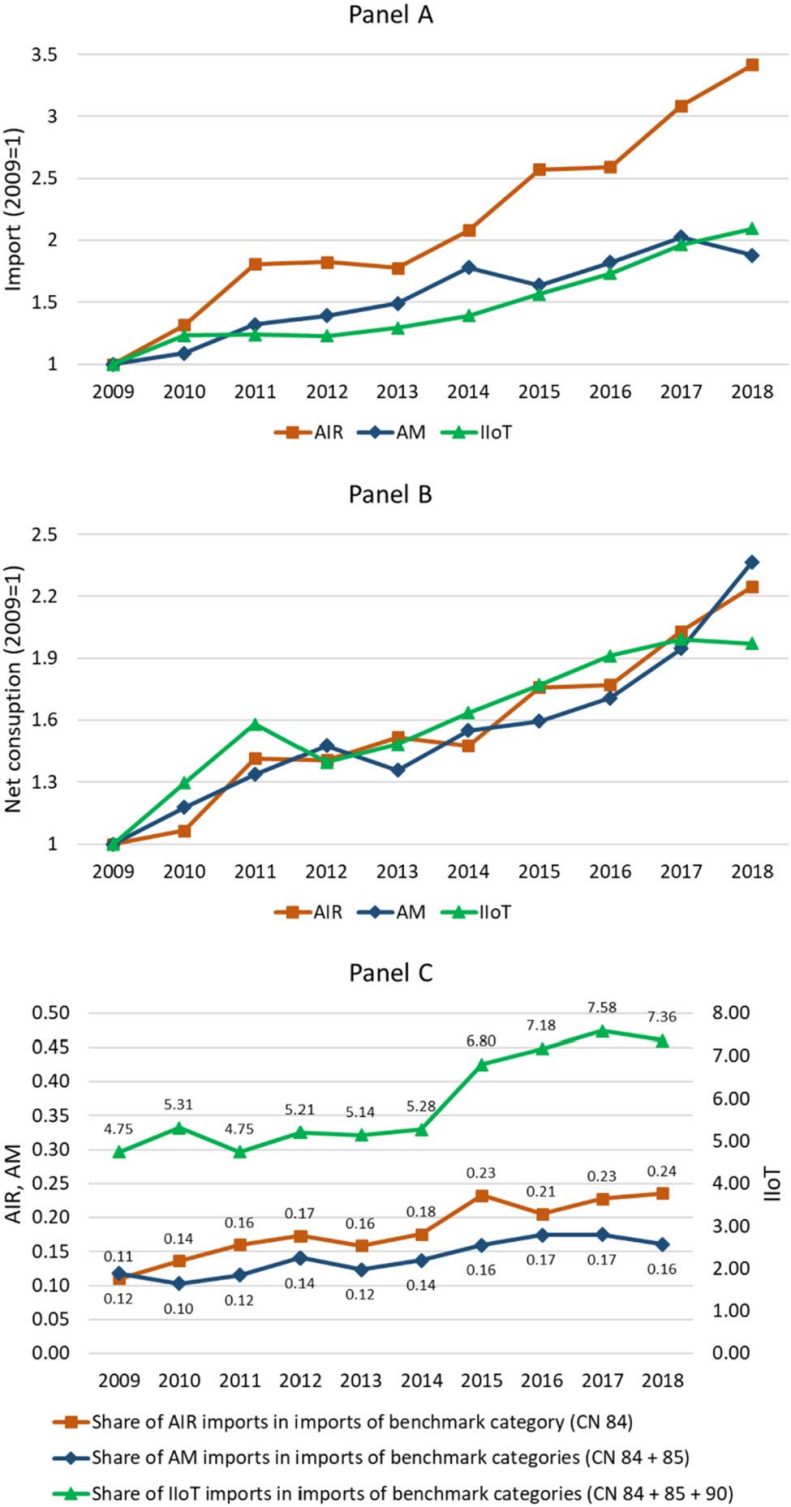
Change in *import* and *net consumption* measures of AMT adoption and shares of AMT imports in imports of the reference benchmark categories (%)—sample of AMT producers in the EU28 sample, 2009–2018 period. Panel **A** reports *import* measures converted in constant PPP USD and reported per 1,000 workers. Panel **B** reports *net consumption* measures converted in constant PPP USD and reported per 1,000 workers. Panel **C** reports the share of imports of each AMT in imports of the reference benchmark categories (%); 2-digit benchmark categories are product category 84 for AIR, the sum of product categories 84 and 85 for AM and the sum of product categories 84, 85 and 90 for IIoT. Given the high level of aggregation characterising our benchmark product categories in the CN classification, reconstructing similar benchmark codes from the CPA classification using the methodology presented in Sect. 3 would result in extensive overlapping and the creation of a high number of synthetic codes (resulting from the aggregation of hundreds of 8-digit CPA product codes), in turn not enabling the computation of a precise benchmark. Source: Comext and PRODCOM databases

Figure 2 explores the change in the flow of *import* (panel A) and *net consumption* (panel B) measures between 2009 and 2018, in the aggregate of the European countries for which we have production data (those for which we can compute the *net consumption* measure). The Figure reports shares of *import* and *net consumption* per 1,000 workers to account for differences in country size; we express them as an index (2009 = 1). Panel A reveals that the adoption of all three AMTs has increased between 1.9 and 2.5 times, with a peak in the *import* proxy for AIRs that reached a 3.5-fold increase. The observed pattern looking at the *net consumption* adoption proxies (panel B) is quite similar, although revealing a more homogeneous growth across the three AMTs, in the aggregate of EU28 countries (i.e. adoption increasing by between 2 and 2.4 times).

Foster-McGregor et al. (2019) highlight that while there has been a rise in the 4IR technologies over the last two decades, the share of these products in total imports remains very small, actually declining over time. To test our measures to this prior finding, Panel C in Fig. 2 reports the shares of the AMT *import* measures in import of reference benchmark categories. As a benchmark, we use the aggregate of the 2-digit product category(ies) in the CN classification to which our product codes (for each AMT) belong (i.e. product category 84 for AIRs, the sum of product categories 84 and 85 for AM and the sum of product categories 84, 85 and 90 for IIoT). Specifically, here we compare AMT imports with imports of similar and related, yet highly aggregated, goods; this allows to avoid confounding effects due to trends in import flows of goods that are completely unrelated with AMTs. When compared with the product category(ies), we observe that all three I4.0 technologies have experienced a trend of increasing shares of imports over the period 2009–2018 relative to their benchmark, with AIRs increasing from 0.11 to 0.24% (+ 114%), AM imports rising from 0.12 to 0.16% (+ 36.9%) and IIoT increasing from 4.75 to 7.36% (+ 55.1%).

As an additional robustness check, Fig. 4 in Appendix B replicates the analysis in Fig. 2, but looking at the full sample of all EU28 countries: panel A explores the change in the flow of *import* measure for our three AMTs between 2009 and 2018, while panel B analyses the trend in the ratio between imports in each AMT and imports of the related benchmark category(ies). Also in this case, as compared to 2009, all three AMTs have increased consistently, with AM and IIoT rising by 1.5 and 2 times, and AIRs even peaking at about 4.9 times. Looking at the share of AMTs in imports of the related benchmarks, the trend is very similar to that observed for the smaller sample of EU countries for which we can compute the *net consumption* measure, with all AMTs increasing their import components in the benchmark categories (AIRs rising by 126.8%, AM by 36.5% and IIoT increasing by 53%).

To provide further insight, in Appendix B, we analyse the composition of the observed trend for AIRs, AM and IIoT, by looking at the shares of aggregate imports across all EU28 countries in single product codes, included in each of our adoption measures. Specifically, Table 8 reports shares for each year in the observation period and product code composing *import* measures for AM and IIoT, as well as the observed percentage change between 2009 and 2018 (for AIRs, Table 8 reports the same data presented in panel B of Fig. 4 since the measure includes a single CN/CPA product code). Such analysis provides insights about some heterogeneity in the trends for individual product codes building our adoption measures, but overall in the vast majority of specific product codes, imports have grown faster compared to the related 2-digit benchmark category(ies), thus leading to an increase in the shares. Specifically, in the case of AM, 5 out of 8 product codes experience an increase in their shares of import (between + 11.8% and + 84.8%), while only 3 product codes experience a slight drop (between – 16.1% and – 19%); similarly, in the case of IIoT, 11 out 13 product codes feature an increase in their share of imports relative to the benchmarks (ranging between + 8.3% and + 225.7%).

Tables 2, 3 and 4 provide detailed data on cross-country differences in the importance of *import* and *net consumption* flows per 1,000 workers of AMTs in 2009 and 2018 (AIRs, AM and IIoT, respectively), as well as their growth over this period.

**Table 2:** *Import* and *net consumption* of AIRs by European country and growth rates between 2009 and 2018

	Import			Net consumption		
	2009	2018	Growth rate (%)	2009	2018	Growth rate (%)
	(1)	(2)	(3)	(4)	(5)	(6)
Austria	9.6	22.3	133.7	19.5	35.1	79.8
Belgium	10.9	21.5	97.7	12.0	7.4	− 38.3
Bulgaria	2.5	6.1	148.8			
Croatia	1.2	6.3	424.5	1.4	3.4	147.5
Cyprus	0.6	0.0	− 98.2			
Czech Republic	4.0	53.9	1262.2	2.7	55.7	1950.6
Denmark	4.3	10.2	138.6	5.6	5.4	− 3.6
Estonia	1.6	4.3	162.4			
Finland	2.9	8.5	198.2	4.8	0.3	− 93.1
France	2.1	6.7	226.0	17.1	25.2	47.6
Germany	4.7	14.6	211.9	11.9	33.4	180.2
Greece	0.8	1.5	86.6			
Hungary	4.2	17.6	321.9	9.6	31.7	231.3
Ireland	1.0	4.5	326.2			
Italy	4.3	12.8	201.9	20.9	69.0	230.7
Latvia	1.4	2.5	76.9			
Lithuania	2.4	7.2	203.9	2.0	3.0	49.4
Luxemburg	16.7	204.5	1123.0			
Malta	6.4	3.9	− 38.8			
The Netherlands	8.5	18.8	121.6	0.6	12.9	1916.0
Poland	2.2	10.9	387.6			
Portugal	4.6	21.4	363.9	1.8	18.2	904.3
Romania	4.3	10.3	137.8			
Slovakia	8.0	73.6	817.4			
Slovenia	5.5	35.8	556.9			
Spain	2.8	9.1	221.8	3.8	9.5	150.5
Sweden	4.3	13.0	198.6	25.7	7.0	− 72.9
United Kingdom	1.8	3.1	74.7	3.0	2.9	− 3.3
All Countires Avg	4.4	21.6	390.0	8.9	20.0	124.7

*Import* and *net consumption* measures converted in constant PPP USD and reported per 1,000 workers

*Source*: Comext and PRODCOM databases

**Table 3:** Import and net consumption of AM by European country and growth rates between 2009 and 2018

	Import			Net consumption		
	2009	2018	Growth rate (%)	2009	2018	Growth rate (%)
	(1)	(2)	(3)	(4)	(5)	(6)
Austria	21.0	31.9	51.7	63.6	123.7	94.5
Belgium	13.6	27.2	99.5			
Bulgaria	9.9	25.8	161.4	0.5	17.6	3359.3
Croatia	19.0	24.8	30.2			
Cyprus	7.9	4.4	− 44.5			
Czech Republic	19.3	54.5	183.2	23.7	38.8	63.6
Denmark	4.0	11.3	183.5	3.7	10.9	190.7
Estonia	8.0	14.9	86.1	0.7	1.9	162.8
Finland	17.1	6.7	− 60.5	14.9	6.6	− 55.5
France	5.9	8.4	40.7	5.6	5.7	0.9
Germany	5.8	15.9	172.3	19.9	67.3	238.4
Greece	10.1	10.8	7.1			
Hungary	7.6	43.5	469.6	0.2	41.5	21,835.6
Ireland	5.4	16.2	196.8			
Italy	7.2	13.5	88.5	33.5	71.1	112.1
Latvia	11.4	33.0	188.7			
Lithuania	10.0	50.2	401.1			
Luxemburg	27.1	11.0	− 59.4			
Malta	15.8	4.9	− 68.8			
The Netherlands	5.5	13.8	152.6			
Poland	12.4	27.7	123.2	6.6	24.8	275.4
Portugal	10.5	24.3	132.0	3.9	7.1	79.5
Romania	13.5	23.9	77.5			
Slovakia	42.3	47.2	11.7	0.4	7.2	1940.3
Slovenia	18.8	65.8	250.1			
Spain	6.7	11.3	67.0	7.3	11.8	60.8
Sweden	4.6	6.8	46.4	0.3	2.1	698.5
United Kingdom	3.1	4.5	46.0	1.5	2.7	76.5
All Countires Avg	12.3	22.7	84.6	11.7	27.5	136.4

*Import* and *net consumption* measures converted in constant PPP USD and reported per 1,000 workers

*Source*: Comext and PRODCOM databases

**Table 4:** Import and net consumption of IIoT by European country and growth rates between 2009 and 2018

	Import			Net consumption		
	2009	2018	Growth Rate (%)	2009	2018	Growth Rate (%)
	(1)	(2)	(3)	(4)	(5)	(6)
Austria	341.3	671.0	96.6	64.1	207.5	223.4
Belgium	526.9	644.5	22.3	118.0	69.0	-41.5
Bulgaria	134.5	360.6	168.1	111.7	170.1	52.3
Croatia	136.9	277.7	102.9	66.1	325.9	392.9
Cyprus	94.3	166.6	76.6			
Czech Republic	798.9	2208.9	176.5	34.7	114.7	230.3
Denmark	270.4	591.3	118.7	210.3	349.2	66.1
Estonia	332.3	1154.2	247.4			
Finland	331.3	735.6	122.0	183.5	294.2	60.3
France	225.5	430.4	90.8	155.2	82.1	-47.1
Germany	397.3	939.2	136.4	236.1	382.8	62.1
Greece	84.1	108.0	28.5	48.0	64.5	34.3
Hungary	1324.6	1903.3	43.7	88.0	452.9	414.5
Ireland	626.3	824.8	31.7			
Italy	173.1	276.8	59.9	211.7	277.0	30.9
Latvia	113.2	652.8	476.6			
Lithuania	113.6	361.6	218.4	47.8	137.1	186.7
Luxemburg	742.4	528.9	− 28.8			
Malta	4892.0	1763.2	− 64.0			
Netherlands	1527.9	4224.5	176.5	5.3	36.8	590.7
Poland	297.6	555.6	86.7	49.5	225.5	355.6
Portugal	190.6	402.2	111.0	90.2	101.6	12.7
Romania	179.4	626.0	249.0	113.1	360.9	219.0
Slovakia	534.7	929.9	73.9	124.8	344.3	175.9
Slovenia	269.5	488.4	81.2			
Spain	118.2	257.1	117.6	114.6	224.1	95.4
Sweden	541.4	728.9	34.6	240.0	221.9	− 7.5
United Kingdom	281.4	624.5	122.0	159.4	434.3	172.4
All countires Avg	557.1	837.0	50.2	117.7	232.2	97.2

*Import* and *net consumption* measures converted in constant PPP USD and reported per 1,000 workers

Source: Comext and PRODCOM databases

Table 2 shows the *import* value of AIRs in 2009 and 2018. Among the European countries, we can observe the central role played by Germany, Italy, Sweden, and Austria during the period, although some Central and Eastern European countries (CEECs) such as the Czech Republic, Hungary, Slovakia and Slovenia complete the scenario, by imposing themselves as important players in the adoption of AIRs.^10^ The *net consumption* data return a very similar picture to the *import* measure, supporting the strong correlation between the two adoption proxies. Moving to AM, Table 3 shows that the biggest importer is Slovakia, followed by Hungary, the Czech Republic and Slovenia. It is worth highlighting the increasing role of CEECs at the end of the period in the *imports* of AM, underlining the importance of the adoption of advanced technologies in these transition countries. Among the most advanced and industrialised countries in the EU, Germany and Italy present the highest growth rate of AM adoption. Finally, looking at data for the IIoT adoption proxies in Table 4, we can observe a more widespread adoption, based on both the *import* and the *net consumption* data, across Europe. Austria, the United Kingdom (UK), Hungary, Poland, and Romania have registered a substantial increase also in *net consumption*, representing the major consumers of IIoT at the end of the period.

It is worth noting that among the advanced European economies, the UK registers not only the lowest initial values of adoption across technologies but also lower growth rates in terms of *import* and *net consumption*, except for IIoT. On the contrary, countries that report important growth rates over the years are located in Central and Eastern Europe. In particular, these countries emerge as strong AMT adopters not just when looking at our *import* measure (as one would expect), but consistently also when looking at the more precise *net consumption* proxy for adoption.

**Fig. 3:**
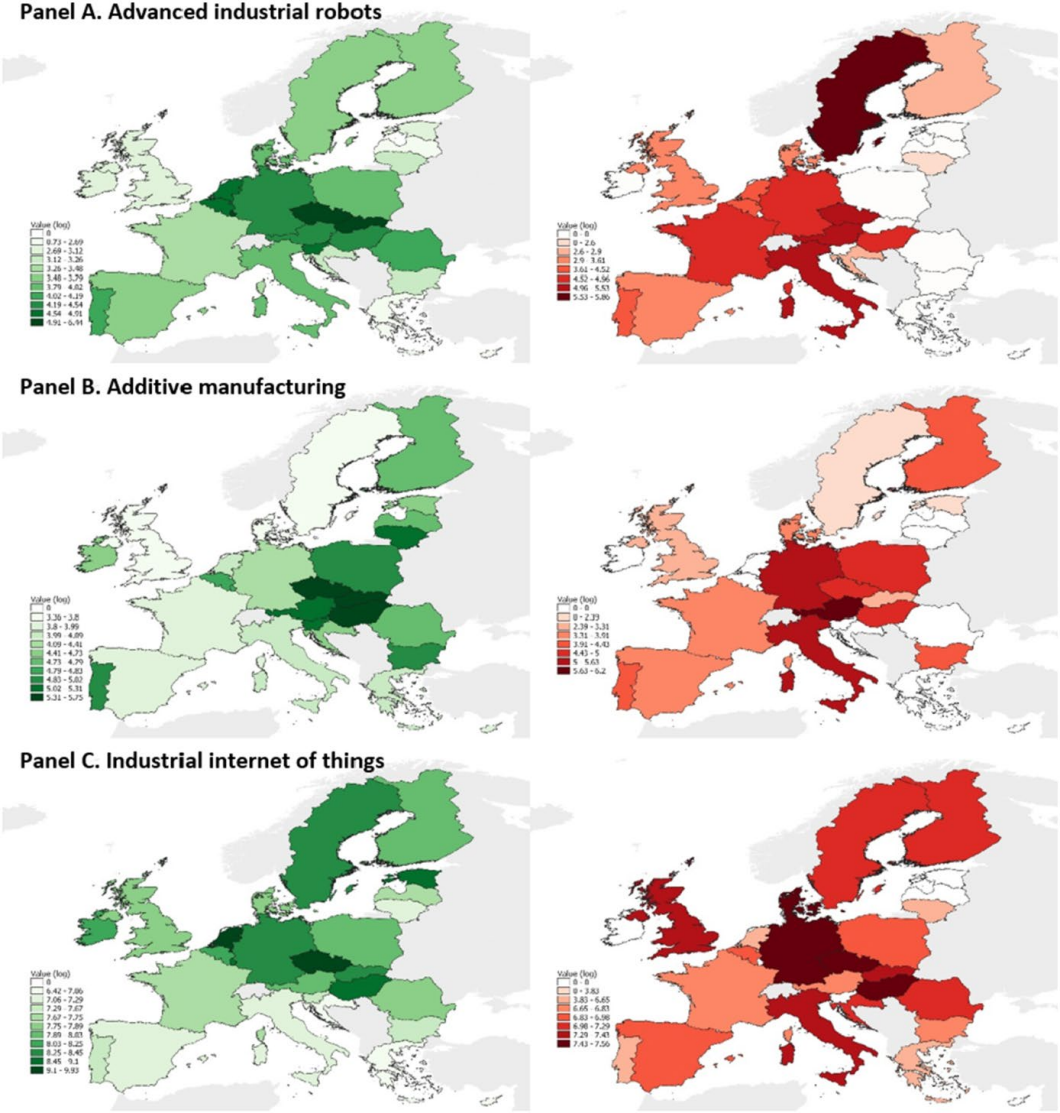
*Import* and *net consumption* of AMTs by EU28 countries, 2009–2018 period stocks. *Notes: Import* (in green) and *net consumption* (in red) measures converted in constant PPP USD, reported per 1,000 workers and expressed as 2009–2018 period stocks (in log). Maps created using QGIS software. Source: Comext and PRODCOM databases

**Fig. 4:**
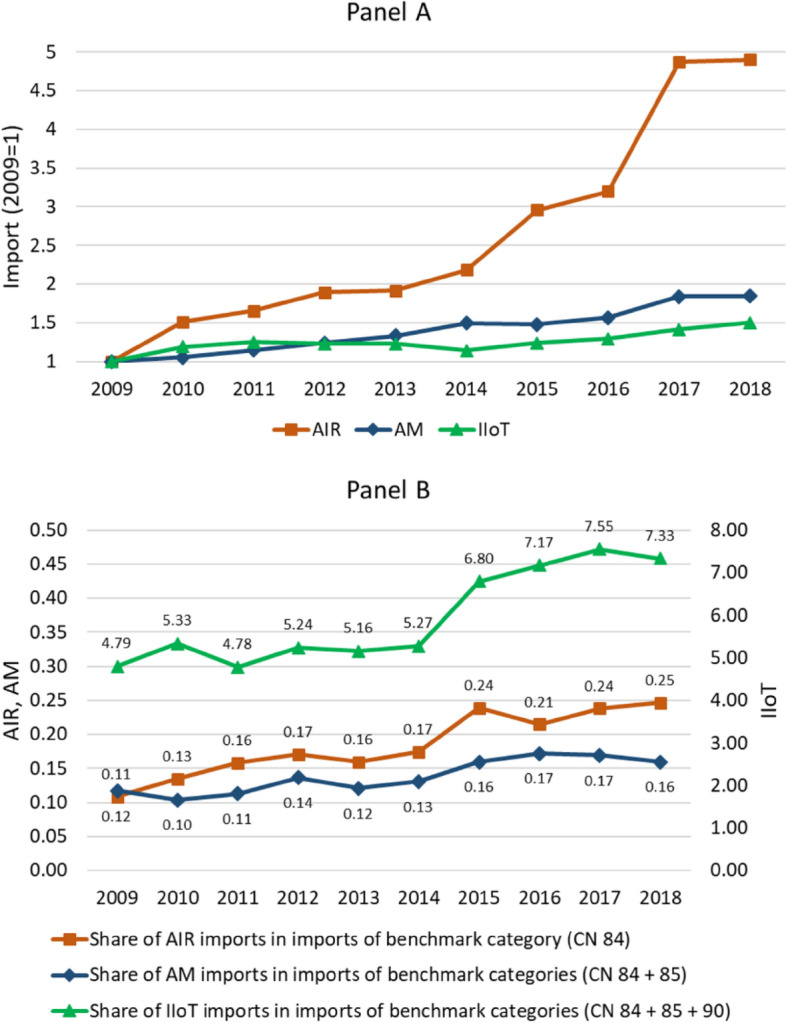
Change in *import* measures of AMT adoption and shares of AMT imports in imports of the reference benchmark categories (%), full sample of EU28 countries, 2009–2018 period. Panel **A** reports *import* measures converted in constant PPP USD and reported per 1,000 workers. Panel **B** reports the share of imports of each AMT in imports of the reference benchmark categories (%); 2-digit benchmark categories are product category 84 for AIR, the sum of product categories 84 and 85 for AM and the sum of product categories 84, 85 and 90 for IIoT. Source: Comext database

In Fig. 3, we further confirm these insights with the cumulated rates of AMT adoption at the end of the period, computed as the stock over the 2009–2018 period per 1,000 workers of both *import* (left-hand side, in green) and *net consumption* (right-hand side, in red) measures. Figure 3 shows the coverage and scale, leaders and laggards in the adoption of AMTs in Europe. The graphical representation makes even clearer the role of Central and Eastern Europe (mainly Hungary, Slovakia, and the Czech Republic) as key adopters, followed by Western European countries such as Germany, Italy, Austria, France and Sweden.

**Fig. 5:**
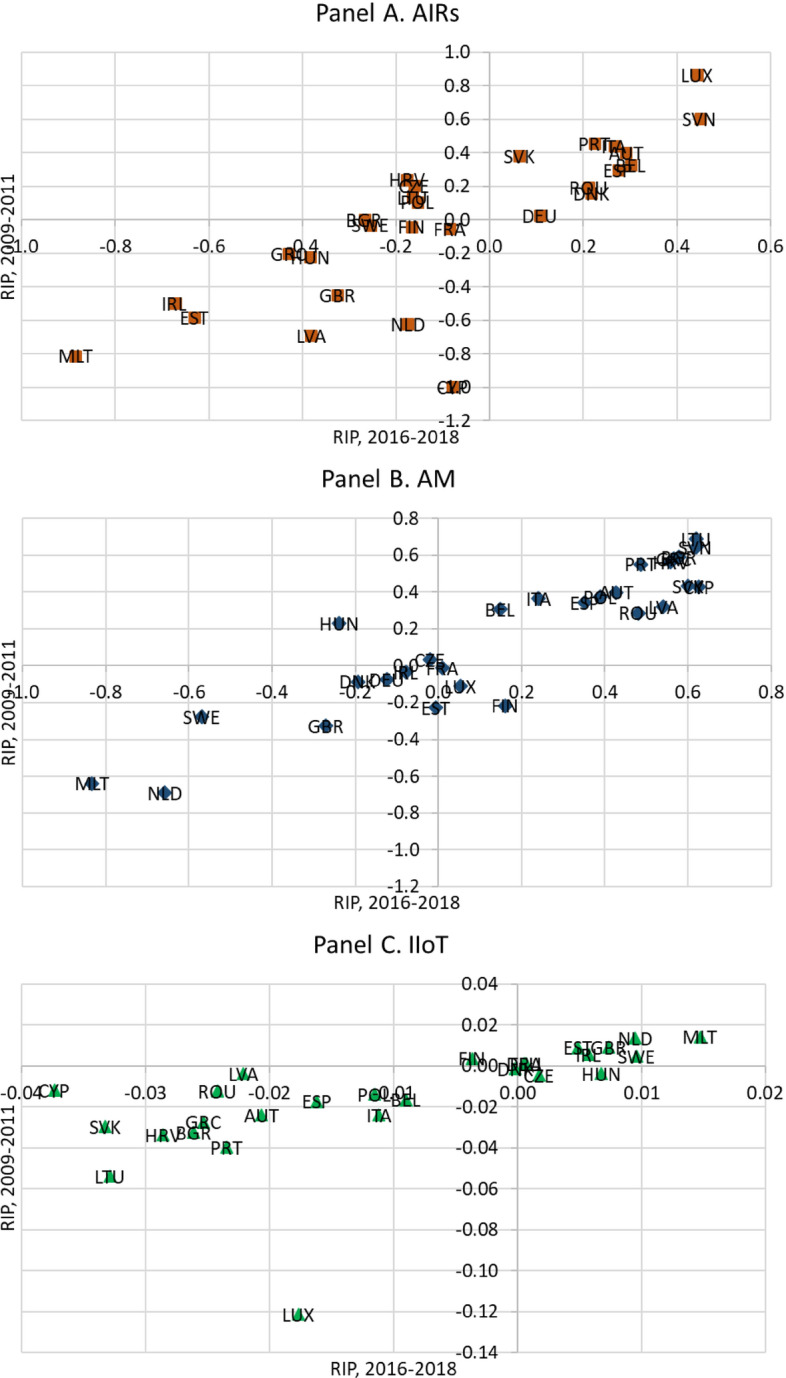
Comparison between the initial and final three-year averages in the relative import propensity (RIP) indexes for each AMT. We compute three-year averages of RIP indexes as simple averages. RIPs for each AMT are transformed as (RIP − 1)/(RIP + 1) to increase symmetry and comparability so that RIPs above 0 indicates indicate a comparative advantage. Source: Comext database

To provide further insight and robustness to our analysis on the adoption and diffusion of I4.0 technologies, we compute normalised relative import propensity (RIP) indexes^11^ in each country and AMT in our sample, following Foster-McGregor et al. (2019). Such complementary analysis provides insight into the evolution of relative intensity in the adoption of each AMT across EU28 countries, at the beginning and the end of our observation period. Results, which are reported in Fig. 5 in Appendix B, denote remarkable stability in the propensity to import AMTs across countries, but with a handful of countries, mostly among the new member states, that have significantly increased their propensity to import AIRs (e.g. Croatia, Czech Republic, Lithuania and Poland) and AM (Hungary).

Two main factors can help explain this pattern. On the one hand, governments of these countries are strongly supporting the investment in the adoption of such *game-changing* technologies given the industrial composition of their manufacturing industries. For example, the Czech Republic is one of the most industrialised countries, where the automotive industry has an important weight in the industrial composition.^12^ Investing in these technologies is crucial for maintaining the (international) competitiveness of the country and for the long-run economic growth, as part of future innovation strategies and industrial policy objectives (Ministry of Industry & Trade of the Czech Republic, 2019).

On the other hand, over the last two decades, CEECs have massively strengthened the link with Western European countries through global value chain participation. At the end of 2005, Western European firms were responsible for around 80% of foreign direct investment (FDI) stock in CEECs, with Germany, Austria, France and Italy accounting for the majority of shares (ECB, 2013). The large-scale investment flow directed from Western European countries towards several CEECs over the last 10–15 years is, in fact, the result of their economic transition from planning and control economies into market economies over the 90s, combined with the benefits of the European Single Market integration policies, as a result of their access to the EU in 2004 (Cséfalvay, 2020). Furthermore, countries like the Czech Republic, Hungary, Slovakia and Poland are the preferred host locations, especially due to their relatively higher political and institutional stability, the availability of relatively skilled workers and the low unit labour costs (Carstensen and Toubal, 2004).

On a complementary perspective, Western European countries are the main destinations of CEEC total exports, 45% related to foreign value-added or domestic value-added for the exports of other countries, suggesting that their participation in GVCs is mostly associated with western (particularly European) multinational enterprises (ECB, 2013, 2020). A strong interdependence with parent firms allows the transfer of sophisticated machinery and capital goods to local affiliates through imports, able to boost productivity upgrading and develop a domestic industry operated by major productive firms in the sector (Chiacchio et al, 2019). Seen under this light, our evidence points at Europe to be the perfect case to understand how MNEs organize and reconfigure the geographical structure of their supply chains over time—for instance, from global to regional, nearshoring activities in CEECs (Pavlínek, 2018)—and how this can have implications also relatedly to the adoption of new technologies.^13^ Recent evidence from Cséfalvay (2020) on AIRs confirms this to be one of the critical factors driving the diffusion of I4.0 technologies across CEECs.

In sum, these findings provide first evidence of the geographical pattern and scale of AMT adoption in Europe: while the most advanced countries have been steadily investing in these technologies in the whole period, we uncover the growing importance of CEECs as I4.0 adopters. At the same time, together with the descriptive statistics provided in Sect. 4.1, our findings provide additional evidence that our *import* and *net consumption* measures return consistent results, with the major advantage of the *import* adoption proxy of being available for an enlarged sample of countries.

## Conclusions, future developments and applications

This paper proposes a fine-grained methodology to measure the adoption of AMTs using trade and production data and provides some descriptive evidence on the patterns of adoption over the last decade across EU countries. Our findings suggest the importance of further investigating the topic and intensifying research efforts to find better, more refined and precise measures able to proxy the adoption of these new technologies. In this respect, the methodology presented in this paper outlines a potential way of overcoming data limitations associated with technologies like AIRs, AM and IIoT. The use of highly disaggregated and detailed trade and production data seems to hold promising opportunities to fill a knowledge gap and offer a powerful tool to investigate how these AMTs are affecting several economic aspects in developed countries, as well as developing countries. Our methodology is easily scalable and can provide up-to-date information on the adoption of AMTs across countries and over time. In particular, considering that the production of AMTs is highly geographically concentrated in a few countries,^14^ in the vast majority of countries imports represent a perfect proxy of adoption. This means that our analysis can be easily extended using 6-digit UN Comtrade data, which are available for all countries in the world and updated regularly to enlarge the sample with non-European countries. Furthermore, while the focus of this paper is at a macro-level, trade data are available at the sector and, increasingly, at the establishment level. Indeed, several statistical offices worldwide are allowing researchers to access detailed import and export data at the transaction level. This opens up the opportunity to build measures of the adoption of AMTs at the firm/establishment level, which so far have been hampered by a chronic lack of information. From a policy perspective, we provide evidence on the adoption and diffusion of AMTs across countries within the European region in a relatively large time window, especially considering countries that are linked through the participation to GVCs orchestrated by western European countries, and the industrial strategies targeting these technologies adopted by CEECs. Data can also provide suggestions and be used to investigate statistically robust causation of the effectiveness of policy incentives put in place to stimulate the adoption of such technologies across countries.

Our effort can provide a set of insights and help define a further research agenda. There are several research areas in the context of I4.0 adoption still under-investigated that can be undertaken using the methodology proposed in this paper.

*Productivity, occupation and growth.* The transition to a digital economy may boost the competitiveness of a country, create new opportunities for business and entrepreneurial initiatives, as well as a new way to reach international markets, affecting productivity and economic growth as a consequence (UNCTAD, 2017, 2020). The manufacturing sector is still recognised as crucial and remains one of the main drivers of employment and economic growth. For this reason, national and supra-national institutions should devote their effort to incentivising and supporting ‘digital development’ investments (Davies, 2015; European Commission, 2017), also monitoring the returns and response to incentives already in place. As existing evidence suggests, new digital manufacturing technologies can boost productivity and sustain GDP growth (e.g. Dauth et al., 2018; Edquist et al., 2019; Graetz & Michaels, 2018). This can be particularly important for emerging economies and their catching-up process, since the adoption of digital technologies may facilitate access to production means and the creation of local (new) enterprises and entrepreneurial initiatives, to contribute to sustainable country development and international competitiveness. However, such technologies can asymmetrically contribute to the growth process, since some countries can have easier access and the ability to use some technologies (e.g. additive manufacturing) rather than others (e.g. advanced industrial robots), due to their peculiar characteristics. Furthermore, these technologies require high-skilled labour (especially with science, technology, engineering, and mathematics (STEM) education). As a form of knowledge-intensive, skill-biased technologies, these could affect occupation, education system, job profile and labour rewards (Frey & Osborne, 2017). Digitalisation may change jobs, their nature and tasks, the skills required and new jobs may emerge as a result of a digital revolution (Brynjolfsson & Mitchell, 2017). This may affect the employment patterns and the demand for skills associated with both existing and new jobs (Grundke et al., 2018). Thus, policy interventions should also operate to create the necessary skills and capabilities to promote and support such digital transition, properly mixing economic and social policy actions to balance potentially rising inequality and managerial control over the workforce (Cetrulo & Nuvolari, 2019).

*International business and global value chains*. Nowadays, companies require more operational flexibility, reduced time-to-market and closer proximity to their consumption markets to be more responsive to local tastes. This may result in the need of reshaping the organisation of global networks and location advantages toward shorter GVC configurations. The higher capital-intensive nature of these digital and automated technologies can change the landscape of country competitive advantages, since the location of manufacturing facilities in low labour-cost countries becomes less and less attractive (Laplume et al., 2016). Besides, these peculiar characteristics may affect the dynamics and drivers of inward/outward FDI, MNEs' internationalisation strategy and location decisions for different value chain activities, and in turn, this may affect GVC organisations (Castellani et al., 2021; Hannibal & Knight, 2018; UNCTAD, 2017). Following this argument, the adoption of AMTs can incentivize the *reshoring* of manufacturing operations—i.e. relocation decision back to the firm’s home country (Kinkel & Maloca, 2009; Ellram et al. 2013) – especially when the company aims at increasing its productivity and flexibility (Dachs et al., 2019), or at enhancing the quality of manufactured products, brand recognition and post-sales processes (Ancarani et al., 2019). Thus, sound empirical evidence can help with the development of effective policies and incentives to boost the digital transformation and influence inward and outward FDI flows. In this respect, the intra-firm co-location of production and Research and Development (R&D) activities is considered crucial to facilitate knowledge transfer across units within the firm’s network and to enhance innovation capabilities, especially when the knowledge is tacit and hard to codify (Pisano & Shih, 2012). However, AMTs can make some knowledge-intensive and production-related research activities more codified and standardised, therefore easy to be transferred across value chain activities and borders. As a consequence, this could affect national and international location and co-location decisions, and the concentration/dispersion of R&D activities and collaboration across places (Castellani & Lavoratori, 2020).

*COVID-19 and current challenges.* The unprecedented disruptions created by the COVID-19 pandemic have strongly challenged businesses across countries and highlighted how sensitive to external shocks particularly dispersed GVCs are, as well as how difficult the management of global organisational structure can be. Recently, the picture has been fuelled by the global shortage of critical components across industries (e.g. semiconductors), and the huge increase in shipping costs per container (Forbes, 2021; UNCTAD, 2021). This has revived the conversation about GVC configurations and more “regionalised” global networks, and how automation and digitalisation can speed such restructuring process, although the sticky nature of GVCs needs to be considered (The Antràs, 2021; Economist, 2020). Furthermore, the COVID-19 shock has caused a “wake-up call” for late digital adopters and the need to start rethinking their operational strategies and business models (Amankwah-Amoah et al., 2021; McKinsey, 2021). Understanding how single AMTs can respond to specific challenges, and whether such technologies can help firms to be more resilient and agile in the long run becomes crucial to create incentives aiming at stimulating timely investment and speeding recovery. Finally, the pandemic has accelerated the call for more environmental-friendly production processes and sustainable manufacturing, where global warming and higher environmental pollution are ascribable to traditional manufacturing technologies, therefore AMTs can play a pivotal role (Bai et al., 2020). In the years to come, rich and up-to-date data are necessary to address all these open questions, and trade data can provide invaluable help in this regard.

## Appendix A: Data validation

In order to validate the selection process for the CN codes reported in Table 1, we consulted a pool of experts composed of both scientists and practitioners, whose expertise relates to the technologies under investigation, as well as to both trade and customs procedures through which CN codes are assigned to capital goods when they are shipped. Overall, the large majority of the 8-digit codes originally identified were confirmed, hinting at the goodness of the overall identification procedure.

As a first step, we checked if and which product codes are used in practice when goods related to our three AMTs of interest are shipped from their producers to clients worldwide. Clearly, CN codes—as any other national or international trade classification—are only used when the shipment of goods involves a cross-border transaction. Conversely, domestic transactions are not recorded on trade registers. Bearing this in mind, we created a survey aimed at confirming the 25 8-digit CN product codes matching with our keywords list and collecting information on any other code used in practice. We sent the survey to 229 worldwide producers of industrial robots, additive manufacturing/3D printing machines, and industrial IoT and automation equipment on the 3rd of June 2020, followed by a first reminder sent on the 10th of June and a final reminder on the 17th of June. Respondents were asked to select one or more technologies associated with products in their catalogues and report which CN codes they use when they export abroad. To maximize the response rate and coverage, as well as provide respondents with the widest range of options, we allowed respondents to choose among other major classifications used in the accounting of both trade and production statistics, alongside the CN classification.^15^ Unfortunately, despite the response period overlapped with the ease of lockdown measures following the COVID-19 pandemic and with many firms starting back their operations, the response rate was heavily penalised as only 3.5% of the firms surveyed completed the questionnaire. Nonetheless, the few answers collected allowed us to validate two product codes associated with our AMTs: the 8-digit CN code 84795000 covering the trade of industrial robots and the 8-digit Prodcom code 28413471, corresponding to the CN code 84639000 and supposedly capturing one of the processes related to additive manufacturing. A further takeaway from the survey came from conversations with a few respondents, carried out via email exchange, who highlighted scarce familiarity with the trade and production nomenclatures we suggested as options in the survey.

As a second step, we consulted practitioners and experts working for the Italian Customs Agency (Agenzia delle Dogane e dei Monopoli) and a private customs broker and logistic service provider.^16^ Phone conversations with these experts helped clarify the steps through which CN product codes are assigned to goods when these transit customs in or outbound. Specifically, goods are classified under a unique classification (e.g. CN, SITC, BEC, etc.) code that describes the product and not its use or specific function. Since incorrect classification can lead to delays in clearing goods, unnecessary overpayment or potential underpayment of duties (the latter resulting in penalties for the shipping firm), this procedure is generally carried out with the highest care and the high majority of firms trading abroad relies on custom brokers to determine the correct product code to be used. In principle, if the shipped good belongs to a very specific category, univocally defined by a product code in the classification adopted, there is little space for errors during the matching procedure. Conversely, in cases where the classification is not up to date with newly developed goods, the identification of the correct product code can suffer from potential misallocations. As custom operators are generally not experts of products, machinery or equipment specificities, when doubts arise, the matching is performed taking the 8-digit product code whose description is the most similar to the in- or outbound good, a more general 6- or 4-digit code, or even the one corresponding to the lowest custom duty among the range of potentially appropriate product codes.

In our specific case, further phone calls with these experts validated other product codes initially selected and associated with our AMTs, upon consultation of private databases to which we could not otherwise get access. Specifically, this second consultation unambiguously validated the selected CN product code for industrial robots (i.e. 84795000) and several codes presumably related to industrial IoT (i.e. all 8-digit codes shortlisted and included in the 4-digit categories 8471, 8526 and 9032), thanks to the high specificity of capital goods associated to these technologies. Furthermore, some procedures were also recorded for the 8-digit code belonging to categories 8517 and 8542, even though only some of them were univocally related to IoT applications. We nonetheless deem these codes validated for the purpose of our investigation, as we are not interested in the actual percentage of matches for each product code in relation to a specific AMT, but rather in confirming that a specific product code was indeed used at least once in trade related to these technologies. From our understanding, the validation of product codes linked to industrial robots and industrial IoT capital goods was possible thanks to the high specificity of good descriptions in both shipment orders and the CN classification, allowing for an unambiguous match in the majority of cases.

The case of additive manufacturing/3D printing machines is relatively more complex as specific CN product codes do not exist yet (as described above). In this specific case, our phone conversation with the custom experts highlighted a lack of expertise on the technology specificities illustrated in Sect. 2, which guided our initial selection. In turn, only one additional product code associated with additive manufacturing was validated (i.e. CN code 84778099), having got confirmed records of trade of 3D printers under this code. Finally, further conversations with one of the experts also confirmed the potential goodness of the selected 8-digit codes 85158010 and 85158090 upon fit with the specific additive manufacturing process for which they were initially shortlisted, however, we could not get confirmation of any custom practice specifically using these codes in relation to shipments of 3D printers using powder bed fusion processes (e.g. laser sintering, laser metal deposition, etc.).

## Appendix B: Additional Tables and Figures

See Tables 5, 6, 7, 8 and Figs. 4, 5

**Table 5:** List of Keywords Related to AMTs

*Keywords related to AMTs*		
Advanced industrial robots		
Robot*	Industrial robot*	
*Additive manufacturing*		
First tier keywords (General terminology, processes, technologies)		
Additive manufacturing	Additive process*	3d print*
3-d print*	3-dimensional print*	3d manufacturing
3-d manufacturing	3-dimensional manufacturing	Three-d print*
Three-dimensional print*	Three-d manufacturing	Three-dimensional manufacturing
Binder jetting	Direct energy deposition	Material extrusion
Material Jetting	Powder bed fusion	Sheet lamination
Vat photopolymerization	Fused deposition modelling	Fused filament fabrication
Laser sintering	Laser melting	Direct metal laser deposition
Laser metal deposition	Electron beam melting	Laser engineering net shaping
Stereolithography	Poly-jet matrix	Multi-jet modelling
Continuous liquid interface production		
Second tier keywords (Components, tools, methods, materials)		
Laser*	Electron beam*	Plasma
Extrusion	Extruder*	Metal*
Plastic*	Resin*	Photopolymer*
Wax*	Powder*	
*Industrial internet of thighs*		
First tier keywords (General terminology, acronyms, concepts, technologies)		
Internet of thing*	IoT	Industrial Internet of thing*
IIoT	Industrial Internet	Communication network*
Automatic network*	Wireless network*	Controller area network
CAN	Public switched telephone network	PSTN
Local area network	LAN	Wireless local area network
WLAN	Long-term evolution network	LTE
Digital subscriber line	DSL	Second generation network
2G	Third generation network	3G
Fourth generation network	4G	Fifth generation network
5G	Next generation network	NGN
Ethernet	Wi-Fi	Advanced sensor*
Automatic sensor*	Advanced actuator*	Automatic actuator*
Advanced communication system*	Automatic regulator*	Automatic controller*
Integrated circuit*	System on chip	SOC
Microcontroller	MCU	Radio-frequency identification
RFID	Near-field communication	NFC
Global positioning system	GPS	Switcher*
Router*	Gateway*	Cable*
Optical fibre*	Data processing machine*	
Second tier keywords (Components, tools, methods)		
Communication*	Network*	Automatic
Sensor*	Actuator*	Reception*
Transmission*	Transfer*	Regulator*
Regulating	Controller*	Controlling
Processor*	Converter*	Amplifier*
Integrated chip*	Chip*	Circuit*
Identifier*	Transponder*	Tag*
Microchip*	Card*	Reader*
Radar*	Navigation*	Switching
Routing	Wire*	Connector*
Optical fiber*	Data	

Keyword selection based on the engineering literature, terminology from ruling bodies and product catalogues on AMTs

**Table 6:** List of initially identified CN product codes related to AMTs

*4-digits HS product codes, 8-digits CN product codes and CN product descriptions*		
Advanced industrial robots		
8479	Machines and mechanical appliances having individual functions, not specified or included elsewhere in this chapter	
	84795000	Industrial robots, not elsewhere specified or included
Additive manufacturing		
8463	Other machine tools for working metal or cermets, without removing material	
	84639000	Other machine tools for working metal or cermets, without removing material; Other
8477	Machinery for working rubber or plastics or for the manufacture of products from these materials, not specified or included elsewhere in this chapter	
	84772000	Extruders
	84775980	Other machinery for moulding or otherwise forming; Other; Other
	84778011	Machines for the manufacture of foam products; Machines for processing reactive resins
	84778019	Machines for the manufacture of foam products; Others
	84778099	Other machinery; Other; Other
8515		Electric (including electrically heated gas), laser or other light or photon beam, ultrasonic, electron beam, magnetic pulse or plasma arc soldering, brazing or welding machines and apparatus, whether or not capable of cutting; electric machines and apparatus for hot spraying of metals or cermets
85158010		Other machines and apparatus; For treating metals
85158090		Other machines and apparatus; Other
Industrial internet of things		
8471	Automatic data-processing machines and units thereof; magnetic or optical readers, machines for transcribing data onto data media in coded form and machines for processing such data, not elsewhere specified or included	
	84718000	Other units of automatic data-processing machines
	84719000	Other
8517	Telephone sets, including telephones for cellular networks or for other wireless networks; other apparatus for the transmission or reception of voice, images or other data, including apparatus for communication in a wired or wireless network (such as a local or wide area network), other than transmission or reception apparatus of heading 8443, 8525, 8527 or 8528	
	85176200	Machines for the reception, conversion and transmission or regeneration of voice, images or other data, including switching and routing apparatus
8526	Radar apparatus, radio navigational aid apparatus and radio remote control apparatus	
	85269120	Radio navigational aid apparatus; Radio navigational receivers
	85269200	Radio remote control apparatus
8542	Electronic integrated circuits	
	85423111	Processors and controllers, whether or not combined with memories, converters, logic circuits, amplifiers, clock and timing circuits, or other circuits; Goods specified in note 9(b)(3 and 4) to chapter 85; Multi-component integrated circuits (MCOs)
	85423119	Processors and controllers, whether or not combined with memories, converters, logic circuits, amplifiers, clock and timing circuits, or other circuits; Goods specified in note 9(b)(3 and 4) to chapter 85; Other
	85423190	Processors and controllers, whether or not combined with memories, converters, logic circuits, amplifiers, clock and timing circuits, or other circuits; Other
	85423911	Other; Goods specified in note 9(b)(3 and 4) to chapter 85; Multi-component integrated circuits (MCOs)*
	85423919	Other; Goods specified in note 9(b)(3 and 4) to chapter 85; Other*
	85423990	Other; Other
9032	Automatic regulating or controlling instruments and apparatus	
	90321020	Thermostats; Electronic
	90321080	Thermostats; Other
	90322000	Manostats
	90328100	Other instruments and apparatus; Hydraulic or pneumatic
	90328900	Other instruments and apparatus; Other

The reference CN classification is the 2017 version. Product codes denoted with *have been checked through assessment of the related additional notes

**Table 7:** Import to export ratios for each AMT, by country and time period

	AIR		AM		IIoT	
	2009–2011	2016–2018	2009–2011	2016–2018	2009–2011	2016–2018
Austria	0.51	0.44	0.45	0.24	1.06	0.93
Belgium	2.61	1.41	1.90	1.77	1.43	1.10
Bulgaria	11.87	1.25	8.68	4.36	1.98	1.21
Croatia	9.36	0.79	2.62	0.82	1.83	2.21
Cyprus			157.50	1.35	10.14	9.59
Czech Republic	2.39	10.72	1.42	1.08	1.28	1.38
Denmark	0.80	0.17	0.35	0.31	1.53	1.48
Estonia	3.00	2.23	2.13	5.10	1.47	0.54
Finland	0.38	0.48	1.31	2.72	1.61	1.46
France	0.60	0.42	1.10	1.15	0.98	0.88
Germany	0.64	0.61	0.12	0.15	1.01	1.05
Greece			5.48	2.88	5.59	4.83
Hungary	0.66	2.21	1.86	6.64	1.29	0.92
Ireland	7.65	3.76	2.56	3.27	0.40	0.23
Italy	0.81	0.50	0.14	0.15	1.67	1.76
Latvia	4.98	2.65	2.03	1.66	1.64	0.82
Lithuania	2.81	1.77	2.27	1.73	2.05	1.27
Luxemburg	0.85	0.61	39.69	10.14	1.05	1.74
Malta			59.73	75.58	32.16	0.47
The Netherlands	0.99	0.73	0.73	0.76	0.91	0.91
Poland	16.80	15.94	3.50	4.16	4.44	2.08
Portugal	1.23	3.17	3.75	7.18	7.60	1.63
Romania	2.51	4.72	7.07	7.79	3.59	1.97
Slovakia	8.63	16.02	0.67	0.55	3.61	2.16
Slovenia	3.92	2.73	1.94	1.86	1.14	1.33
Spain	1.50	1.23	1.62	2.01	4.30	2.91
Sweden	0.21	0.29	0.71	0.99	1.26	1.31
United Kingdom	1.54	1.09	0.88	1.35	1.42	2.17

We compute three-year averages of import to export ratios as simple averages. Ratios above 1 indicates net importers of AMTs, conversely ratios below 1 indicates net exporters of AMTs

**Table 8:** Shares of CPA/CN product codes imports in imports of the reference benchmark categories (%) and changes between 2009 and 2018 (%), full sample of EU28 countries

		2009	2010	2011	2012	2013	2014	2015	2016	2017	2018	∆(2009–2018)
AIR	CPA 28993935/CN 84795000	0.109	0.135	0.158	0.171	0.159	0.175	0.239	0.215	0.238	0.247	126.8
AM	CPA 27903181/CN 85158010	0.010	0.009	0.007	0.009	0.008	0.007	0.010	0.011	0.009	0.009	-16.1
	CPA 27903191/CN 85158090	0.019	0.017	0.018	0.022	0.019	0.020	0.024	0.025	0.026	0.024	30.2
	CPA 28961030/CN 84772000	0.023	0.020	0.024	0.023	0.021	0.022	0.030	0.031	0.030	0.029	26.0
	CPA 28961075/CN 84775980	0.010	0.008	0.010	0.010	0.010	0.010	0.010	0.011	0.009	0.009	-16.4
	CPA 28961082/CN 84778011	0.002	0.001	0.001	0.002	0.002	0.001	0.002	0.003	0.002	0.002	11.8
	CPA 28961084/CN 84778019	0.006	0.005	0.004	0.004	0.004	0.003	0.005	0.005	0.004	0.005	-19.0
	CPA 28961097/CN 84778099	0.037	0.033	0.040	0.047	0.045	0.057	0.065	0.071	0.075	0.069	84.8
	Synthetic CPA for 28413471, 28491360, 289900Z0/CN 84639000	0.010	0.009	0.010	0.019	0.012	0.011	0.014	0.016	0.014	0.014	38.6
IIoT	CPA 26113003/Synthetic CN for 85423111, 85423119	0.121	0.102	0.079	0.102	0.094	0.066	0.071	0.125	0.107	0.152	25.5
	CPA 26113006/CN 85423190	0.971	1.110	0.995	1.036	0.972	0.974	1.211	1.307	1.530	1.475	51.9
	CPA 26113091/Synthetic CN for 85423911, 85423919	0.020	0.036	0.033	0.024	0.023	0.025	0.029	0.030	0.059	0.065	225.7
	CPA 26113094/CN 85423990	0.729	1.000	0.803	0.839	0.830	0.863	1.082	1.073	1.153	1.141	56.6
	CPA 26302320/CN 85176200	1.617	1.769	1.635	1.887	1.947	2.054	2.865	3.066	3.097	3.021	86.8
	CPA 26,512,050/CN 85269120	0.309	0.266	0.237	0.242	0.224	0.240	0.298	0.300	0.294	0.258	-16.3
	CPA 26512080/CN 85269200	0.028	0.029	0.032	0.040	0.041	0.045	0.054	0.056	0.056	0.052	89.8
	CPA 26516500/CN 90328100	0.013	0.017	0.020	0.022	0.024	0.027	0.032	0.041	0.046	0.039	198.8
	CPA 26517015/CN 90321020	0.028	0.029	0.029	0.031	0.032	0.033	0.044	0.049	0.049	0.045	63.3
	CPA 26517019/CN 90321080	0.050	0.050	0.047	0.050	0.048	0.048	0.055	0.057	0.050	0.045	-9.5
	CPA 26517030/CN 90322000	0.024	0.026	0.029	0.034	0.031	0.029	0.034	0.036	0.034	0.036	51.0
	CPA 26517090/CN 90328900	0.332	0.371	0.361	0.408	0.393	0.380	0.449	0.455	0.454	0.404	21.6
	Synthetic CPA for 26122000, 26203000, 269900Z0/Synthetic CN for 84718000, 84719000	0.552	0.527	0.481	0.521	0.499	0.489	0.574	0.579	0.622	0.598	8.3

We take the CN-CPA correspondence in 2017 as the reference. Whenever a change in the classifications impacts the identified product codes in 2017, we highlight any data manipulation with '*Synthetic*', following the methodology by Van Beveren et al. (2012). Any other change in the classifications which is related to the years before and after 2017 (e.g. a change in the product code identifying a specific product category) are not highlighted here. 2-digit benchmark categories are product category 84 for CPA/CN codes related to AIR, the sum of product categories 84 and 85 for CPA/CN codes related to AM and the sum of product categories 84, 85 and 90 for CPA/CN codes related to IIoT

## Footnotes

1 Several initiatives from national governments have taken place worldwide, starting with the ‘Advanced Manufacturing Partnership’ in the USA and the ‘High-tech Strategy 2020’ in Germany, followed by ‘La Nouvelle France Industrielle’ in France, the ‘Future of Manufacturing’ in the United Kingdom, ‘Industria 4.0’ in Italy, the ‘Factories of the Future’ as part of the European Programme Horizon 2020 (Liao et al., 2017; Mariani and Borghi, 2019); some, emerging also in developing countries like Morocco (Gallab et al., 2021).

2 See Dachs et al. (2019) for a recent application.

3 Data included in the Comext data set are converted in Euros for reporting purposes by Eurostat and expressed in current prices.

4 For instance, in Comext, quantities are usually reported in 100 kg, while in PRODCOM are reported in different units such as kg, m2 or number of items.

5 Given its wider discussion in the existing literature (e.g. Acemoglu and Restrepo, 2018), the identification of the relevant nomenclature is a lesser problem in the case of AIRs; hence, as a reference, we looked at ABB Ltd product catalogue. Concerning AM, we looked at product catalogues from three main producers worldwide, namely Stratasys Ltd., 3D Systems Inc. and EOS GmbH. Finally, relatively to IIoT, given that it is the technology bearing the widest set of capital goods among the technologies we focus on, we consulted product catalogues from Intel Corp., ABB Ltd., Siemens AG, Hewlett Packard Enterprise LP, Bosch GmbH, GE Digital Plc., Cisco System Inc. and Rockwell Automation Inc.

6 For more details, see https://trade.ec.europa.eu/tradehelp/classifying-computers-software.

7 We define the product categories of interest starting from CN-2017 classification (that following the latest revision of the HS classification, HS-2017).

8 See also Keller (2004) for an extensive review.

9 As a further robustness check, since virtually all European countries in our sample (with the exception of Cyprus, Greece and Malta in the case of AIRs) are also exporters of AMTs—similarly to what found by Caselli and Coleman (2001) in the case of computing equipment already in the mid-90s—we computed import to export ratios for each country and each technology in order to show which countries are net importers of AMTs (ratios above 1) and which countries are exporters of AMTs (ratios below 1). To show the evolution of this dynamics over the observation period we computed initial (2009–2011) and final (2016–2018) three-year averages, to smooth potential peaks in the data. We report this additional analysis in Table B3 in Appendix B. The vast majority of countries in our sample (19 in the case of AM, 20 in the case of IIoT and 14 in the case of AIRs) consistently import more AMTs than they export.

10 We suggest caution in the interpretation of values for Cyprus, Malta, and Luxemburg.

11 These indexes are positive (negative) if the share of imports more of a certain AMT in total import of a given country is higher (lower) than the corresponding share in the EU as a whole.

12 After the global financial crisis in 2009, car manufacturers worldwide started to restructure their business operations, investing heavily in new digital technologies. For instance, since 2010 the automotive industry has witnessed rising investments in new production capacities as well as investments in modern production technologies, resulting in major car-producing countries driving the demand for industrial robots (IFR, 2020).

13 The latest data from Eurostat seems to corroborate our evidence, highlighting that, across European countries, AMT adoption is mostly concentrated in large firms. In 2020, across EU27 countries, only 4% of small enterprises (10–49 employees) employ AIRs, while this share grows to 23% among large enterprises (250 + employees). Similarly, these shares amount to 4% and 17% in the case of AM, to 16% and 38% in the case of IIoT, respectively for small and large firms (Eurostat, 2021).

14 Our production data indicates that, even within the sample of countries featuring some AMT production (i.e. those reported in the scatterplots in Fig. 1), the large majority of EU production is concentrated in few countries, particularly in the case of AIRs (mostly Austria, Denmark, France, Germany, Italy, Hungary and Sweden) and AM (mostly Austria, Czech Republic, Denmark, Germany, Italy and Slovakia). Conversely, production of IIoT is much more evenly distributed across all EU producers.
